# From Human Monocytes to Genome-Wide Binding Sites - A Protocol for Small Amounts of Blood: Monocyte Isolation/ChIP-Protocol/Library Amplification/Genome Wide Computational Data Analysis

**DOI:** 10.1371/journal.pone.0094164

**Published:** 2014-04-14

**Authors:** Sebastian Weiterer, Florian Uhle, Sabin Bhuju, Michael Jarek, Markus A. Weigand, Marek Bartkuhn

**Affiliations:** 1 Department of Anesthesiology and Intensive Care Medicine, Justus-Liebig-University Giessen, Germany; 2 German Centre for Infection Research (DZIF), partner site Giessen-Marburg-Langen, Giessen, Germany; 3 Genome Analytics, Helmholtz Centre for Infection Research, Braunschweig, Germany; 4 Institute for Genetics, Justus-Liebig-University, Giessen, Germany; CRCL-INSERM, France

## Abstract

Chromatin immunoprecipitation in combination with a genome-wide analysis via high-throughput sequencing is the state of the art method to gain genome-wide representation of histone modification or transcription factor binding profiles. However, chromatin immunoprecipitation analysis in the context of human experimental samples is limited, especially in the case of blood cells. The typically extremely low yields of precipitated DNA are usually not compatible with library amplification for next generation sequencing. We developed a highly reproducible protocol to present a guideline from the first step of isolating monocytes from a blood sample to analyse the distribution of histone modifications in a genome-wide manner. Conclusion: The protocol describes the whole work flow from isolating monocytes from human blood samples followed by a high-sensitivity and small-scale chromatin immunoprecipitation assay with guidance for generating libraries compatible with next generation sequencing from small amounts of immunoprecipitated DNA.

## Introduction

Epigenetic regulatory mechanisms like covalent histone modifications and DNA methylation mediate changes in gene expression without changing the underlying DNA sequence. In the first case histones can be posttranslationally modified on their N-terminal tails by enzymes with different kinds of modifying qualities, resulting in acetylated, methylated or phosphorylated amino acid residues. Previous studies showed that the specific presence or absence of such histone modifications in defined genomic, especially cis-regulatory regions like promoters is correlated with the expression of associated genes. For example it has been clearly demonstrated, that trimethylation (me3) of lysine (K)4 and an acetylation (ac) of K9 of histone H3 are associated with transcriptionally active or at least poised promoter regions. In contrast a trimethylation of H3K27 can be found at repressed loci [1],[2],[3],[4].

Hence, an analysis of epigenetic parameters can help understanding gene regulatory mechanisms underlying gene expression programs. In addition, studying these epigenetic parameters in cells affected by disease may reveal new findings about unknown pathophysiological mechanisms [5],[6].

ChIP (chromatin immunoprecipitation) is the method of choice to analyze the occurrence of histone modifications and transcription factors in a native chromatin context. Since the arrival of high-throughput technologies like tiling microarrays (ChIP-chip) and especially since it is possible to sequence immunoprecipitated DNA in a massively parallel fashion (ChIP-seq) it is possible to obtain precise maps of such epigenetic marks on a genome-wide scale. On the other hand there are still a couple of limitations under certain experimental settings that hamper an easy and reproducible workflow [7]. These are majorly dependent on the requirements for the amounts of ChIP-DNA that have to be used with the majority of library preparation protocols. This is especially true in case of biological/medical studies that rely on the usage of limited primary cell material, like (in our study) monocytes.

Monocytes are cells of the innate immune system and play a key role in the human body’s defence mechanism. They are derived from hematopoietic stem cells in the bone marrow [8]. After passing through several progenitor stages they terminally differentiate into monocytes, with several scavenger receptors for pathogen recognition. Under inflammatory conditions stimulation can induce the release of mediators to activate subsequent immune response mechanisms [9] [10]. In addition, monocytes have the potential to move via the bloodstream to target tissues in order to differentiate into dendritic cells and macrophages during an infection [11] [12].

Different kinds of cell surface receptors are known, that can classify monocyte subpopulations based on their expression levels (CD14++ CD16-, classical monocytes; CD14+ CD16++ non-classical monocytes; CD14++ CD16+ intermediate monocytes) [13]. The classical CD14++ CD16- monocytes seem to play an important role in different kinds of diseases, like cardiovascular diseases [14], or relevant immune processes, as the initial pro-inflammatory cascade of the innate immune system during sepsis [15]. They also represent a central factor in the impact of immune dysfunction especially dealing with severe bacterial infections [16]. In addition a persistence of the compensatory anti-inflammatory response syndrome is associated with monocyte dysfunction [17]. Although there is a correlation between immunological dysfunction and the monocyte human leukocyte antigen-DR, which can be used as a marker for immunsuppression, the clear pathophysiological structure cannot be determined [18], [19],[20]. Despite all efforts in the immunological field, the exact mechanisms leading to diseases involving pathological changes in monocyte phenotype and monocyte dysfunction are still unclear. A genome-wide epigenetic analysis of CD14++ CD16- monocytes from critically ill patients with different kinds of inflammatory diseases has a great potential to reveal novel pathophysiological mechanisms.

This protocol is a guideline for the experimenter from the first steps of isolating monocytes from a blood sample to the genome-wide analysis of histone modifications via cross-linked ChIP-seq. So far such a protocol for limited amounts of primary blood cells, which would be reproducibly applicable with donors’ or patients’ blood, has not been described in the literature. As a consequence of the limited availability of human blood samples and due to inherent loss of cells during cell purification the starting amount of cells is usually not enough to obtain sufficient amounts of immunoprecipitated DNA to meet the requirements for standard library preparation protocols. Therefore we expanded our protocol and included the steps of a robust library amplification protocol compatible with such low DNA amounts into the workflow. Furthermore, we scaled down our protocol to uncritically small blood sample sizes, which safely can be taken from critically ill patients.

In conclusion, the following protocol describes the complete workflow from isolating monocytes from human blood samples to a high-sensitivity and small-scale chromatin immunoprecipitation assay. In addition instructions for amplification of sequencing libraries from small amounts of immunoprecipitated DNA assures sufficient DNA quantity for performing ChIP-seq and subsequent genome wide computational data analysis.

## Materials and Methods

### Experimental Design (see Fig. 1)

**Figure 1:**
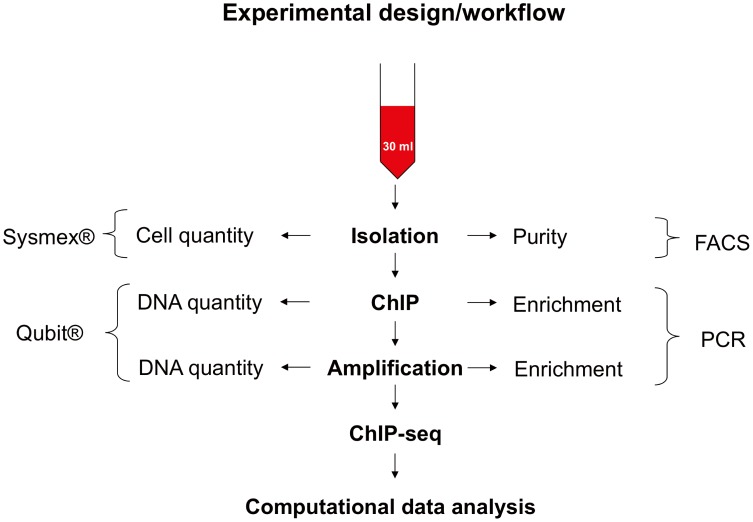
Experimental design/workflow. Diagram shows the experimental design for a complete workflow to analyse genome wide histone modifications of CD 14++ CD 16- monocytes using 30 ml of blood.

#### Monocyte isolation

A blood sample of 30 ml was taken and peripheral blood mononuclear cells were isolated using ficoll-based density gradient centrifugation. Further isolation of CD14++ CD16- monocytes was conducted using magnetic cell sorting to deplete CD16++ monocytes and separate CD14++ monocytes. Purity of isolation was checked by flow cytometric analysis of the CD14+ cell fraction.

#### Cell preparation

Cells were cross-linked for 10 min using formaldehyde (final concentration 1%).

#### ChIP protocol

The ChIP protocol was optimized for small amounts of monocytes. Chromatin has to be sheared using ultrasound sonication. To gain satisfying results, a specialised sonication programme with additional adapted cooling is indispensable. We designed a shortened ChIP protocol, where immunoprecipitation, reverse cross-linking and DNA purification is executed within 2 days.

#### Library amplification

Library amplification was conducted using a specially modified library preparation kit protocol to supervise library amplification with the presence of SYBR Green in a real-time PCR machine.

#### ChIP sequencing

Sequencing of the libraries was performed on an Illumina HiSeq 2000.

#### Computational data analysis

In addition to identification of enriched and depleted genomic regions and annotations, correlation analysis was performed to compare and validate data quality. Furthermore we compared our data to publicly available expression data in order to identify functional relationships between chromatin states and gene expression.

### Ethics Statement

All research was conducted according to the principles expressed in the Declaration of Helsinki, and approved by the ethics committee of the medical faculty of the Justus-Liebig-University of Giessen (Klinikstrasse 32, D-35385 Giessen, Germany) with the approval number 155/12. Subjects provided written, informed consent on forms approved by the Institutional Review Board.

### Reagents

- PBS (1%)
- PBS (1%) +EDTA (2 mM) +BSA (5%)
- CD16 MicroBeads (Miltenyi Biotec)
- CD14 MicroBeads (Miltenyi Biotec)
- FITC anti-human CD14 Antibody (BioLegend)
- Formaldehyde
- Glycine
- Protease inhibitors (Roche complete). Dissolve 1 tablet in 10 ml H_2_O to obtain 10x concentrated stock solution
- Lysis buffer (1% SDS, 10 mM EDTA, 50 mM Tris/HCl, pH8.1, 1x concentrated protease inhibitors)
- Agarose A/G-Beads (CalBiochem IP-05)
- Dilution buffer (SDS 0.01%, Triton X-100 1%, EDTA 1.2 mM, Tris/HCl, pH 8.1 16.7 mM, NaCl 167 mM)
- Low-salt wash buffer (SDS 0.1%, Triton X-100 1%, EDTA 2 mM, Tris/HCl, pH8.1 20 mM, NaCl 150 mM)
- High-salt wash buffer (SDS 0.1%, Triton X-100 1%, EDTA 2 mM, Tris/HCl, pH8.1 20 mM, NaCl 500 mM)
- LiCL wash buffer (LiCl 0.25 M, NP40 1%, Deoxycholat 1%, EDTA 1 mM, Tris/HCl, pH 8.1 10 mM)
- TE (Tris/HCl, pH8.1 10 mM, EDTA 1 mM)
- RNase A
- Proteinase K
- SYBR Green I dye (Life Technologies)
- 80% ethanol
- Ficoll

qPCR primer:

TLR4 forward TGTGTGCCCTGGTTTGTTTA.

TLR4 reverse GCCCCTGTTAGCACTCAAAA.

HMGB1 con1 forward AAGCTGTGTACGGTGTGTGC.

HMGB1 con1 reverse GCCTCCTAAAAAGGTCCTTGA.

### Equipment

- Illustra GFX PCR DNA and Gel Band Purification kit (GE Healthcare Life Sience)
- Sonifier 250 (Branson)
- Qubit Fluorometric Quantitiation (Life Technologies)
- Magnetic cell sorting (autoMACS, Miltenyi Biotec)
- Flow cytometer (e.g. FACSCalibur, BD Bioscience)
- MicroPlex Library Preparation kit (Diagenode)
- Real-time PCR (Bio-Rad CFX96)
- AMPure XP beads (Agencourt)
- Magnetic rack
- Hematology analyzer (Sysmex KX-21N)

### Sequencing and Computational Methods

#### Sequencing

4 barcoded libraries (input, H3K9ac, H3K4me3 and H3K27me3) per donor were prepared and sequenced on a single lane of an Illumina HiSeq 2000. In total we generated data sets for 4 donors. Cluster generation was performed using the Illumina cluster station. Sequencing on the Illumina HiSeq 2000 followed a standard protocol. The fluorescent images were processed to sequences using the Genome Analyzer Pipeline Analysis software 1.8 (Illumina). All sequencing data was deposited at NCBI’s gene expression omnibus (GEO) under the accession number GSE54634.

#### Processing of ChIP-seq reads and peak calling

ChIP-seq reads were converted to fastq format and aligned to a precompiled hg19 reference index with BOWTIE [21]. Unambiguously mapped and unique reads were kept for subsequent generation of binding profiles and calling of peaks using PeakRanger [22] and MACS [23], where the sequencing reads from input DNA were used as control. We applied an FDR-threshold of 0.05. All downstream analyses were done in R/BioConductor (http://www.bioconductor.org). We made use of the following R/BioConductor packages: *GenomicRanges* [24], *GenomicFeatures* [25] and *chipseq* [26].

After extension of reads continuous coverage vectors were calculated and z-standardized to account for differential library sizes. Those coverage vectors were used for visualization in genome browsers as well as to collect data in 5 kb windows spanning the transcriptional start sites of all RefSeq genes. The binding data was binned across binding sites in 50 bp bins and the mean was calculated at each position in order to generate profiles of binding data. K-means clustering was done using seqMINER [27].

#### Identification of enriched/depleted genomic annotations

Annotations (transcriptional start sites (TSS), transcriptional end sites (TES), −10kb to −1 kb upstream of genes (TSS upstream), introns and exons) were derived from human hg19 RefSeq genes downloaded from UCSC genome database. H3K4me3 and H3K27me3 peak ranges were intersected with these intervals and the relative association was calculated and compared to the genomic background.

#### Correlation analysis

We determined the number of reads mapping to the promoters (from−1kb upstream to 1 kb downstream from the transcriptional start site) of all RefSeq genes. After quantile normalization we calculated Pearson’s correlation coefficient for all pair wise combinations of experiments.

#### Public data sets used in this study

In order to obtain gene expression values for all genes in a highly similar cell type we used a pre-aligned (hg19) BAM-file from a CD14+ monocyte long-RNA-seq (ENCODE consortium [28]) downloaded via the UCSC interface:

wgEncodeCshlLongRnaSeqMonocd14CellPamAlnRep1.bam

Reads were imported into R and for each RefSeq gene we determined the number of reads within exons. These count data were normalized by dividing them by the respective gene length. Finally expression values were log2 transformed.

### Procedure

### Monocyte Isolation (Day 1)

Transfer 30 ml blood into a 50 ml tube.
Isolate peripheral blood mononuclear cells via density gradient centrifugation (Ficoll).
Wash the pellet of peripheral blood mononuclear cells (repeat this step 2 times):
  - add room-temperature (RT) PBS (Phosphate-Buffered Saline) (1%) up to 30 ml.
  - centrifuge cells at RT at 1500 rpm for 5 min,
  - remove the supernatant and discard.
4. Add room-temperature (RT) PBS (1%) up to 30 ml, centrifuge cells at RT at 800 rpm for 10 min, remove the supernatant and discard.
Add 2 ml PBS (1%) +EDTA (2mM) +BSA (5%) and resuspend pellet.
Dilute a small amount of the cell suspension 1∶10 (e.g.: 10 µl cell suspension+90 µl PBS) to determine cell number using a hematology analyser (Sysmex) for subsequent antibody calculation.
Centrifuge cells at RT at 800 rpm for 10 min, remove and discard the supernatant.

#### Critical step!

Pure isolation of peripheral blood mononuclear cells at this point is one of the key steps that can affect a successful outcome. On the other hand the recurrent wash steps can cause significant loss of cells.

#### Caution!

The use of blood samples from patients, who had multiple blood transfusions or blood dialysis may lead to impureness of the peripheral blood mononuclear cells gained with the density gradient centrifugation. In addition, also an increased immune reaction may affect purity of the density gradient centrifugation.

### CD16 Depletion

8. Add 50 µl PBS (1%) +EDTA (2mM) +BSA (5%)/5×10^7^ cells+50 µl CD16 MicroBeads (Miltenyi Biotec)/5×10^7^ cells.
9. Mix well and incubate for 30 minutes at 4°C.
10. Wash cells with 2 ml per 10^7^ cells and centrifuge 10 min with 300 g at 4°C. Resuspend with 500 µl PBS (1%) +EDTA (2 mM) +BSA (5%)/10^8^ cells.
11. Proceed with depletion using magnetic cell sorting or manual cell sorting.
12. Dilute a small part of the cell suspension 1∶10 (e.g.: 10 µl cell suspension+90 µl PBS (1%)) to determine cell number.

#### Critical step!

Inexact incubation time may cause unspecific binding and loss of CD14++ CD 16- monocytes.

### CD14 Separation

13. Add 80 µl PBS (1%) +EDTA (2 mM) +BSA (5%)/10^7^ cells+20 µl CD14 MicroBeads (Miltenyl Biotec)/10^7^ cells.
14. Mix well and incubate for 15 minutes at 2–8°C.
15. Wash cells with 1–2 ml/10^7^ cells and centrifuge 10 min with 300 g at 4°C. Resuspend with 500 µl PBS (1%) +EDTA (2 mM) +BSA (5%)/10^8^cells.
16. Proceed with separation using automatic magnetic cell sorting or manual magnetic cell sorting.
17. Dilute a small part of the cell suspension 1∶10 (e.g.: 10 µl cell suspension+90 µl PBS (1%)) to determine cell number using hematology analyser.

#### Critical step!

Disregard of incubation time may cause unspecific binding and loss of purity.

### FACS (Fluorescence-Activated Cell Sorting) Analysis

18. Centrifuge isolated CD14++ CD16- monocytes with 300 g for 10 minutes. Resuspend in 2 ml PBS (1%) at RT.
19. Extract 100 µl cell suspension, add 20 µl antibodies (FITC anti-human CD14 Antibody, BioLegend) and incubate for 30 minutes in the dark at 2–8°C for FACS analysis.

#### Caution!

FACS analysis at this point is obligate to assure pure results and isolation efficiency.

### ChIP (Chromatin Immunoprecipitation)

20. **Cross-link DNA/protein.** Resuspend cells in 2 ml PBS, add 55.6 µl 37% formaldehyde to adjust to a concentration of 1% formaldehyde. Incubate at 18°C for precisely 10 min with gentle shaking.
21. Add 0.125 M final concentration of glycine to stop fixation (136 µl of 2 M glycine in total of 2 ml cross-linking solution) and incubate for 5 min at RT.
22. Centrifuge 5 minutes at 2000 rpm at 4°C, remove the supernatant and discard.
23. Resuspend pellet with 1 ml 4°C PBS (1%). Centrifuge 5 minutes at 2000 rpm at 4°C, remove the supernatant and discard. Cross-linked cells can be stored at −20°c at this point. **1. Potential break time on day 1.**

### Sonication/Shearing Chromatin

24. Add 200 µl lysis buffer to 10^6^ cells in a 1.5 ml tube.
25. Start the sonication with a Branson sonifier to achieve chromatin fragments of 150–600 base pairs. Secure tube using a clamp to ensure a constant tip position in the tube and that the probe does not touch the tube wall. To generate a constant cooling effect, keep the tube on wet ice with ethanol during sonication. Use a duty cycle of 10% and 45 sec “on” pulses and 15 sec “off” pulses. Repeat 20 times resulting in a sonication time of 20 minutes in total.

#### Critical step!

Sonication is one of the most critical steps in this protocol. We highly recommend strict adherence. Our experience using other equipment for sonication did not show reliable results.

#### Caution!

We recommend constant cooling during sonication and strict observance of the “off” pulse time. Skipping the pause time after 45 sec of sonication may not lead to satisfactory results.

26. After sonication, centrifuge tubes for 10 minutes with 14000 rpm at 4°C. From here on: keep samples on ice/at 4°C.
27. Transfer supernatant into fresh tube. You may freeze your sample here!

**2. Potential Break Time on Day 1.**

#### Caution!

For use of multiple samples we recommend sequential processing for the sonication step.

#### Preparation of Immunoprecipiation

28. Add 1800 µl dilution buffer to the tube with 200 µl of sheared chromatin (Dilution 1∶10).
29. Store 38 µl of the dilution buffer chromatin mix in a separate tube at 4°C as an “input” extract (10%).
30. Per sample, add 20 µl agarose A/G-beads (Calbiochem) to 2 fresh tubes (one for binding of antibody, one for pre-clearing of chromatin extract). Add 1 ml dilution buffer, mix and centrifuge for 1 minute with 5000 rpm at RT and discard the supernatant.
31. Antibody binding: Add 500 µl dilution buffer and add appropriate amount of antibody (we used 3 µl) to one of the agarose A/G-beads filled tubes. Incubate at 4°C in a rotator for at least 1 hour, centrifuge for 1 minute with 5000 rpm and discard the supernatant.
32. To pre-clear chromatin add 380 µl of the dilution buffer chromatin mix in the second of the agarose A/G-beads filled tubes. Incubate at 4°C in a rotator for at least 1 hour.
33. Centrifuge for 30 sec with 5000 rpm at 4°C.

#### Immunoprecipitation

34. Transfer pre-cleared chromatin (supernatant) into tubes with antibody-bound agarose A/G-beads (from step 31).
35. Incubate at 4°C in a rotator for at least 3 hours.
36. Centrifuge for 1 minute with 5000 rpm at 4°C and discard the supernatant.

#### Wash Chromatin Bead Complex

37. Add 1 ml of low-salt wash buffer on top of the beads; mix on a rotator for 10 minutes at 4°C and centrifuge at 5000 rpm. Remove supernatant and discard.
38. Add 1 ml of high-salt wash buffer on top of the beads; mix on a rotator for 10 minutes at 4°C and centrifuge at 5000 rpm. Remove supernatant and discard.
39. Add 1 ml of LiCL wash buffer on top of the beads; mix on a rotator for 10 minutes at 4°C and centrifuge at 5000 rpm. Remove supernatant and discard.
40. Add 1 ml of TE wash buffer on top of the beads; mix on a rotator for 10 minutes at 4°C and centrifuge at 5000 rpm. Remove supernatant and discard. Repeat this step one time and resuspend chromatin bead complex in 100 µl TE at RT.
41. Add 62 µl of TE wash buffer to the input sample from step 29 to adjust to the same volume as in the immunoprecipated tubes.

#### Reverse Cross-linking

42. Add 2 µl RNase A (10 mg/ml) to each tube and incubate for 30 minutes at 37°C (optional: mix on a rotator).
43. Add 5 µl 10% SDS and 2,5 µl Proteinase K (20 mg/ml) and incubate for 4 h at 37°C. Shift to 65°C over night!.

#### DNA Purification (Day 2)

Use Illustra GFX PCR DNA and Gel Band Purification kit (GE Healthcare Life Science) and the “protocol for purification of DNA from solution or an enzymatic reaction” to purify ChIP-DNA, according to manufacturers recommendations.

44. Add 50 µl of pure H_2_O for DNA elution. You may freeze your sample here. **1. Potential break time on day 2.**

#### Determination of Library DNA Amount

For ChIP-DNA quantitation we recommend to use the Qubit Assay.

#### qPCR for Validation

#### Critical step!

To ensure that the ChIP was successful, we advice the execution of qPCR on selected target sequences (exemplarily shown in Fig. S3).

#### Library Amplification

To conduct library amplification, use the MicroPlex Library Preparation kit (Diagenode). After steps A. “MicroPlex Template Preparation” and B. “MicroPlex Library Synthesis”, which can be done as described, a variation of the protocol is necessary.

45. After mixing the library amplification buffer, enzyme and water with the library and 2 µl of Indexing Reagent, extract 27 µl of the solution and add SYBR Green I dye (Life Technologies) at a 6x final concentration, and process parallel to the actual tube.
46. Centrifuge the solution and transfer it to a real-time PCR machine (we used a BioRad CFX96 machine). PCR protocol as follows:

Step 1: 1 cycle: 72°C 3 min

Step 2: 1 cycle: 85°C 2 min

Step 3: 1 cycle: 98°C 2 min

Step 4: 4 cycles: 98°C 20 sec; 67°C 20 sec, 72°C 20 sec

Step 5: 4–21 cycles 98°C 20 sec, 72°C 50 sec

#### Caution!

Remove the non-SYBR Green tubes, when SYBR Green tubes show a decreasing acceleration in the exponential curve.

#### Critical step!

When working with multiple samples avoid long breaks in the PCR process while removing none-SYBR Green tubes.

#### Library Purification

Library purification is adapted to existing conditions based on the instruction manual for the MicroPlex Library kit. For library purification use AMPure XP beads.

47. Transfer PCR product without SYBR Green to a low binding tube and add 50 µl of AMPure XP beads. Resuspend AMPure XP by pipette until no pellet is visible.
48. Incubate 5 minutes at RT, then place tube on magnetic rack and wait for 2 minutes until beads are bound completely.

#### Critical step!

We recommend gentle shaking during incubation.

49. Remove tube and proceed further according to manufacturers’ recommendations.
50. For final DNA elution add 30 µl of pure H_2_O.
51. Place tube on magnetic rack and wait for 2 minutes until beads are bound completely.
52. Remove the supernatant without disturbing the pellet and transfer to a fresh low binding tube.

#### qPCR for Validation

#### Critical step!

To validate the amplified product, we advice the execution of qPCR on selected target sequences.

#### Determination of Library DNA Amount

For determination use Qubit Assay to quantify amplified DNA products.

### Results

**Figure 2:**
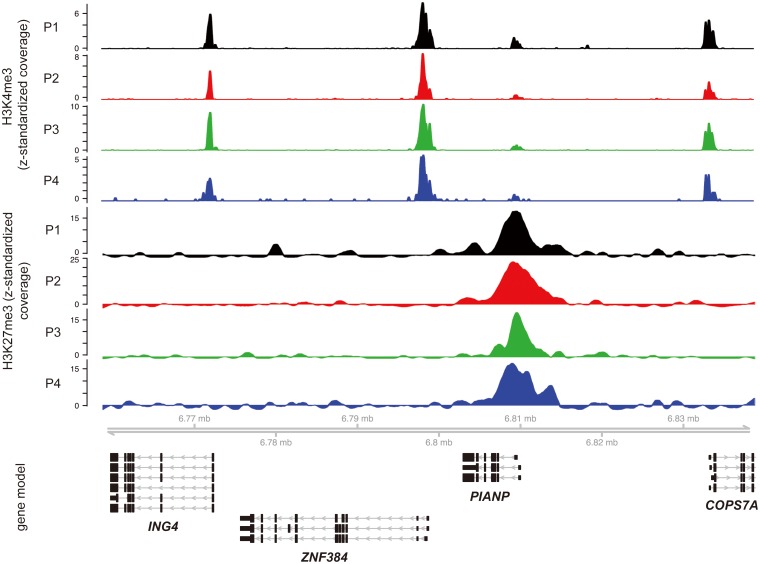
Genome browser snap shot of an 80 kb region on chromosome 12 of hg19. Depicted are the z-standardized ChIP-seq coverage profiles for 2 histone modifications (H3K27me3 and H3K4me3) from 4 blood donors (P1–P4). Strong H3K4me3 binding can be detected around the transcriptional start sites of the *ING4*, *ZNF384* and *COPS7A* genes. These genes are majorly devoid of H3K27me3 signals. In contrast the *PIANP* gene is strongly enriched for H3K27me3 but shows only weak signals for H3K4me3.

In order to show applicability of the approach described above we purified CD14++ CD16- monocytes and subsequently prepared formaldehyde-cross-linked chromatin extracts from 4 healthy blood donors. Purity was monitored by FACS and was in all cases equal or above 95 percent (Fig. S1). For each sample we performed chromatin-immunoprecipitation experiments with antibodies specific for the histone modifications H3K4me3, H3K27me3 and H3K9ac. Whereas H3K4me3 [29] and H3K9ac [30] have been repeatedly shown to be associated with promoters of actively transcribed genes, H3K27me3 is known for its role in polycomb-mediated gene repression [31]. After preparation of barcoded libraries we always sequenced 4 of them on a single lane of an Illumina HiSeq 2000. After read alignment, filtering and quality control inspection of the ChIP-seq data revealed highly similar profiles when comparing the binding data for a given histone modification across the 4 different donors. This is exemplarily shown for H3K4me3 and H3K27me3 data in genome browser snap shots (Fig. 2). Even on this level the high degree of reproducibility becomes instantly visible. Additionally several principal features of the data can be appreciated. Of the four genes in the depicted genomic interval on chromosome 12, three are strongly marked by H3K4me3 with the typical bimodal pattern across the transcriptional start sites of the respective genes (*ING4*, *ZNF384* and *COPS7A*). These genes are virtually free of H3K27me3. In contrast the *PIANP* gene shows only relatively weak association with H3K4me3 but instead is marked by a strong H3K27me3 signal. Similar to H3K4me3, the H3K27me3 signal peaks in the promoter region but is obviously much more spread out which is in line with reports from the literature [4].

**Figure 3:**
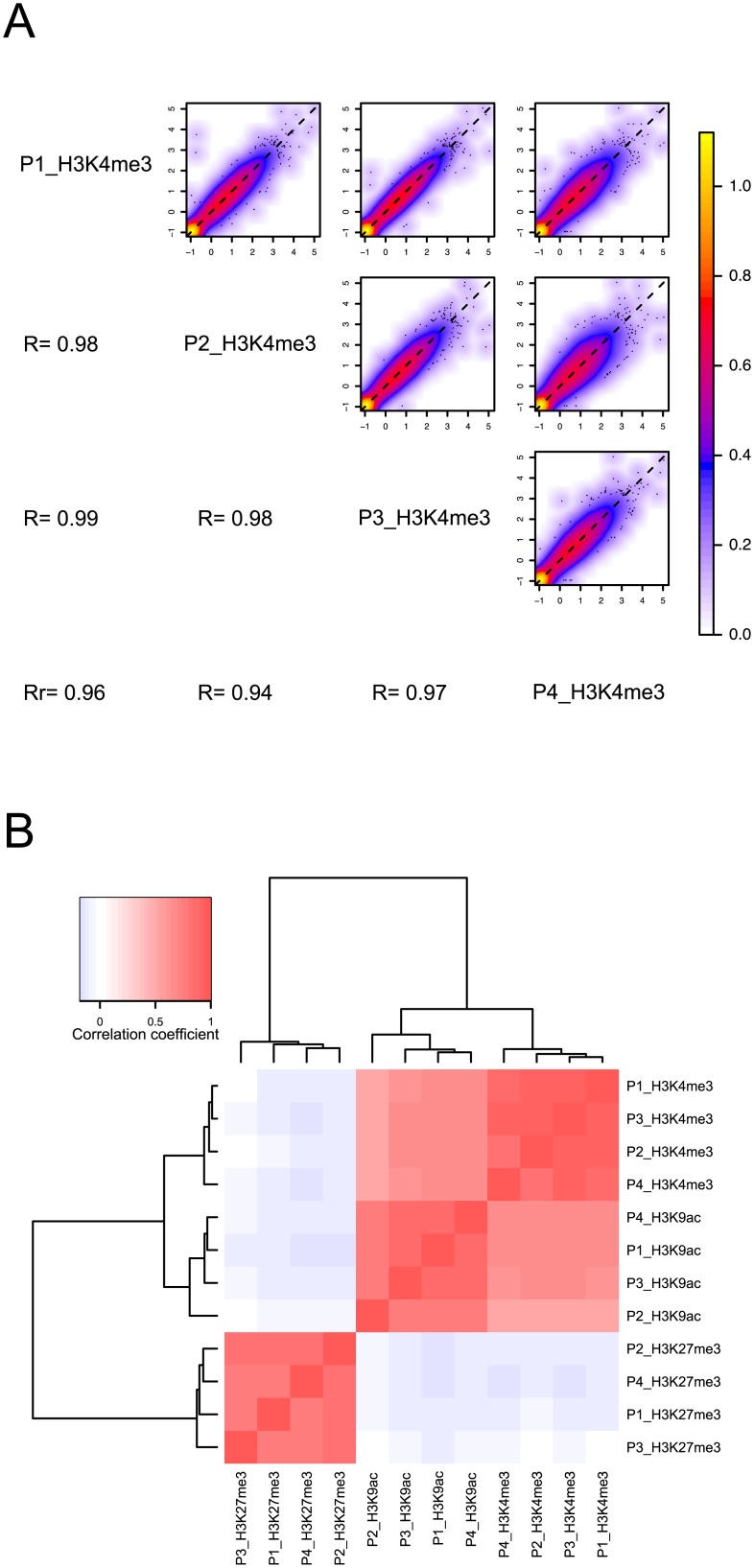
Correlation analysis shows high reproducibility between independent biological samples. A) H3K4me3 binding data from 4 donors was compared against each other with respect to binding to promoters. Quantile-normalized read counts per promoter interval (+/−1 kb around the transcriptional start sites) were plotted for all pair-wise combinations as smoothed scatterplots (color-coding of density is shown in color key). Furthermore Pearson’s R was calculated for each combination. B) Pair wise correlation was done for H3K4me3, H3K27me3 as well as H3K9ac data from 4 donors. The hierarchical cluster analysis of the resulting correlation matrix identifies two major groups: H3K9ac and H3K4me3 are strongly correlated whereas both are negatively correlated with H3K27me3 (color-coding of correlation coefficients is shown in color key).

In order to substantiate the observed similarity between experiments we compared the data for a given antibody on genome-wide scale. Pair wise scatter-plots comparing the promoter association of a given histone mark validate the findings from inspection of profiles in the genome browsers (Fig. 3A). For example we found a mean correlation coefficient R^2^ = 0.97 for H3K4me3 (R^2^_H3K27me3_ = 0.85; R^2^_H3K9ac_ = 0.82; Fig. S2). Additionally, we compared all conducted experiments amongst each other with respect to their similarity. When we did hierarchical clustering of the pair wise correlation coefficients we found the data to segregate into the expected groups. I.e. H3K4me3 and H3K9ac show a high degree of correlation whereas the repressive H3K27me3 mark is clearly different from the two active marks (Fig. 3B).

**Figure 4:**
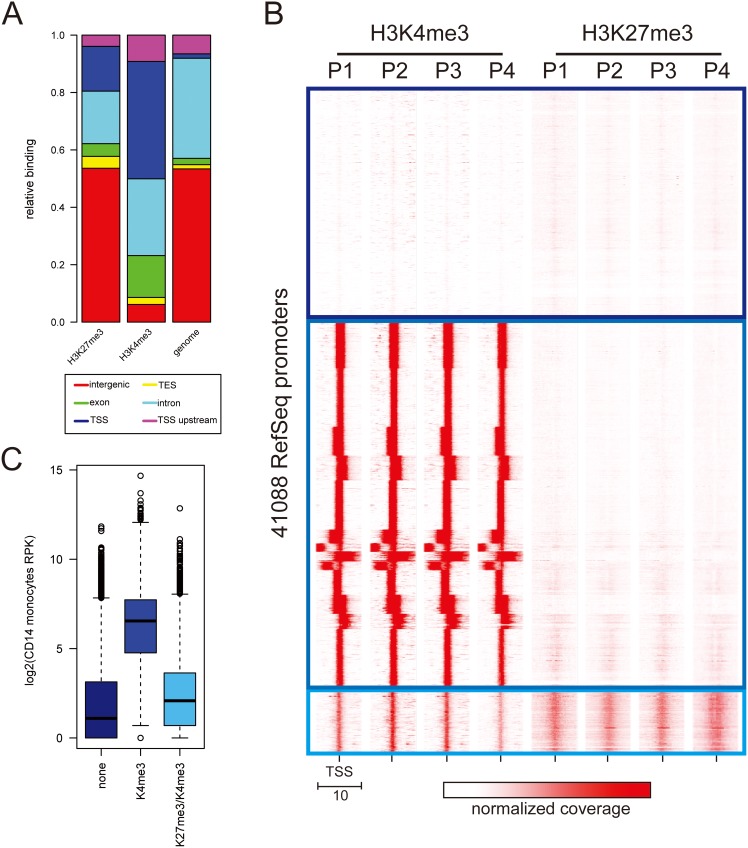
Histone modifications at promoters of CD 14++ CD 16- monocytes. A) H3K4me3 and H3K27me3 are both enriched at promoters. Significantly bound regions were compared with the distribution of genomic features (TSS, TSS upstream, TES, exon, intron, intergenic) based on RefSeq gene annotations. In comparison to the genomic background (genome) both H3K27me3, but especially H3K4me3 are strongly enriched for TSSs. B) K-means clustering reveals 3 major chromatin states at 44109 RefSeq transcriptional start sites. The heat map shows binding (in red) centered across a 10 kb interval spanning all RefSeq TSSs for H3K27me3 and H3K4me3 in 4 blood donors. The data was clustered by k-means which separates the data into 3 major promoter classes: no histone modification (dark blue), high H3K4me3/no H3K27me3 (blue) and low but detectable H3K4me3 and high H3K4me3 (light blue). C) Promoter classes are associated with specific transcriptional output. Gene expression levels in CD14+ monocytes were calculated for all RefSeq genes (based on ENCODE RNA-seq data, shown as reads per 1 kb of transcript length). Gene expression values of genes falling into above mentioned categories are plotted as boxplots.

Optical inspection of the genome browser and the obvious association with promoter regions prompted us to investigate the genomic features associated with regions of significant binding in an unbiased manner. Therefore we compared binding peaks for H3K4me3 and H3K27me3 with the following genomic annotations: transcriptional start sites (TSS and TSS upstream region), transcriptional end sites (TES), exons, introns and intergenic regions. When comparing with the genomic background distribution of these features it became evident that both H3K4me3 as well as H3K27me3 are strongly enriched at transcriptional start sites (Fig. 4A - dark blue bar), although this association is somewhat stronger for H3K4me3. In order to obtain a systematic overview about the binding across all annotated promoters within the human genome, we used the binding profiles for H3K4me3 and H3K27me3 in a 10 kb window around the transcriptional start sites of all RefSeq (Reference Sequence) promoters and performed cluster analysis by k-means. The heat-map representation clearly indicates 3 major classes of promoters (Fig. 4B), those completely devoid of any modification, those high in H3K4me3 and virtually free of H3K27me3 and those with high levels of H3K27me3 and low but detectable H3K4me3. We reasoned that these “promoter chromatin states” might be associated with distinct transcriptional output. Therefore we extracted expression values for all RefSeq transcripts from a recent RNA-seq data set from the ENCODE (Encyclopedia of DNA Elements [32]) consortium obtained from CD14+ monocytes (Fig. 4C). As expected we find the modification free promoters to be expressed at very low levels whereas the genes with strong H3K4me3 binding across the TSS (transcription start site) have significantly higher expression. In contrast bivalently modified promoters are associated with gene expression levels comparable to those of unmodified promoters very similar to what has been described for this specific promoter class [33].

All together these data clearly demonstrate that the method described above is able to generate highly reproducible, high quality genome-wide binding data for a specific cell type isolated from low amounts of blood samples.

## Conclusions

Epigenetic regulation of chromatin displays an important cellular mechanism to control nuclear processes like transcription, replication or DNA repair. Misregulation may be associated with diseases, like tumor growth or cellular dysfunction [34]. In order to understand the underlying mechanisms and the consequences of malfunction it is crucial to study this regulatory system in primary cells derived from human samples. Studying epigenetic mechanisms largely involves the application of methods allowing to determine the chromatin landscape on genome-wide scale, including ChIP-seq. Due to the inherently low amounts of DNA precipitated with ChIP and the limitations with respect to available biological sample material we here suggest a protocol that allows to study chromatin modifications (or binding of chromatin factors) on genome-wide scale in monocytes purified from human blood samples. Importantly, sample volume is low enough in order to be compatible with blood withdrawal from critically ill patients, an important fact with respect to studying the impact of disease conditions on epigenetic gene regulation. Key to the suggested protocol was finding a way to overcome restrictions regarding the expected amount of precipitated DNA relevant for the preparation of sequencing libraries. This was achieved by applying a commercially available solution that allows preparation of libraries from very low amounts of starting material.

Collectively, the method generates highly reproducible binding profiles and therefore allows studying epigenetic mechanisms in low amounts of chromatin derived from human monocytes. The particular cell type has proven to be potentially relevant for disease related processes affecting the human immune response. Nevertheless, the methods should be applicable to other sub-populations of human blood cells, too.

## Supporting Information

Figure S1 **Purity of isolated monocytes as determined by FACS.** FACS experiment with anti-human CD14 antibody shows the efficiency of the isolation after CD16 - PBMC depletion and following CD14++ monocyte separation. To determine the purity of the cell isolation by flow cytometry, initial conservative gating on all cellular events was performed based on forward-/side scatter properties and subsequent determination of CD14+ cellular events within this gate.
(TIF)

Figure S2 **Correlation analysis for H3K27me3 and H3K9ac.** A) H3K27me3 binding data from 4 donors was compared against each other with respect to binding to promoters. Quantile-normalized read counts per promoter interval (+/−1 kb around the transcriptional start sites) were plotted for all pair-wise combinations as smoothed scatterplots (color-coding of density is shown in color key). Furthermore Pearson’s R was calculated for each combination. B) Same as in A) for H3K9ac.
(PDF)

Figure S3 **qPCR for validation of individual ChIP.** Exemplary ChIP-qPCR for one of the analysed donors: analysis of H3K4me3 and H3K27me3 binding to the promoter of the TLR4 gene and a control region of the HMGB1 locus is shown. The TLR4 gene is active in CD14++ CD16- monocytes and accordingly we find strong association of H3K4me3 with the promoter. In contrast no H3K27me3 can be detected. As expected the control region in the HMGB1 locus (HMGB1 con1) is devoid of both histone modifications.
(TIF)
