# Supplementary figures and images for: From Human Monocytes to Genome-Wide Binding Sites - A Protocol for Small Amounts of Blood: Monocyte Isolation/ChIP-Protocol/Library Amplification/Genome Wide Computational Data Analysis
Source: PLoS One. 2014 Apr 14;9(4):e94164. doi: 10.1371/journal.pone.0094164 (PMC3986051; doi:10.1371/journal.pone.0094164)

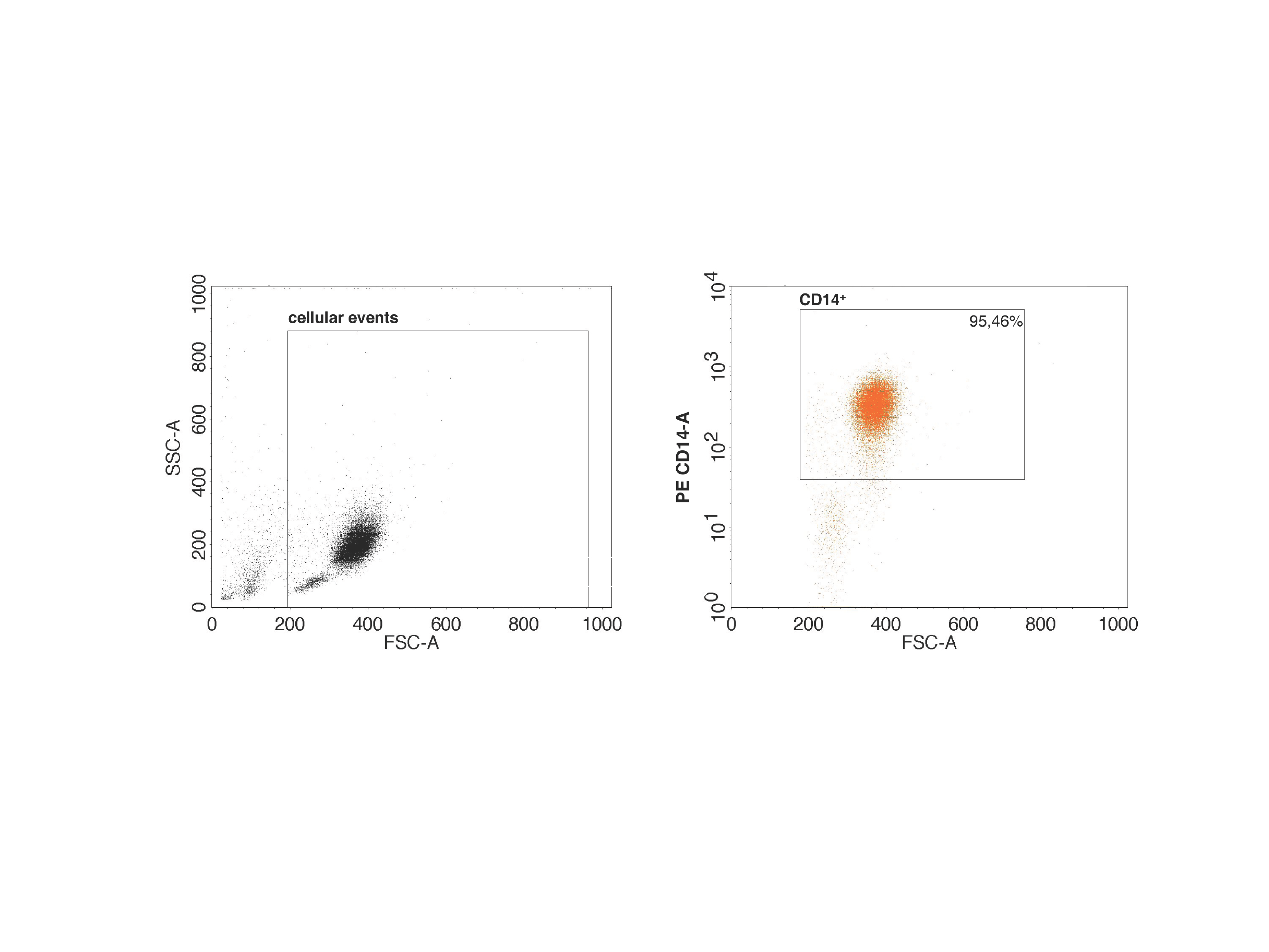

Supplement: Figure S1 — Purity of isolated monocytes as determined by FACS. FACS experiment with anti-human CD14 antibody shows the efficiency of the isolation after CD16 - PBMC depletion and following CD14++ monocyte separation. To determine the purity of the cell isolation by flow cytometry, initial conservative gating on all cellular events was performed based on forward-/side scatter properties and subsequent determination of CD14+ cellular events within this gate. (TIF) [file pone.0094164.s001.tif]

A

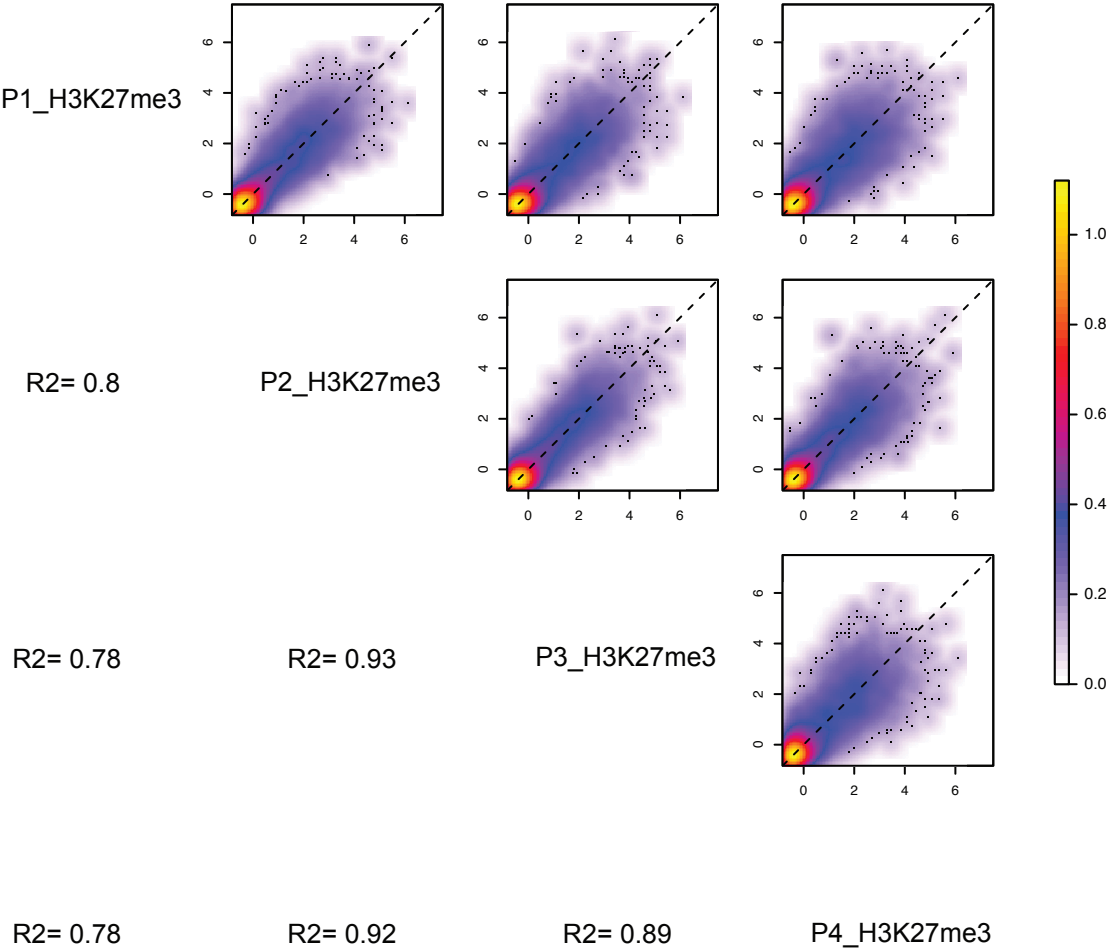

B

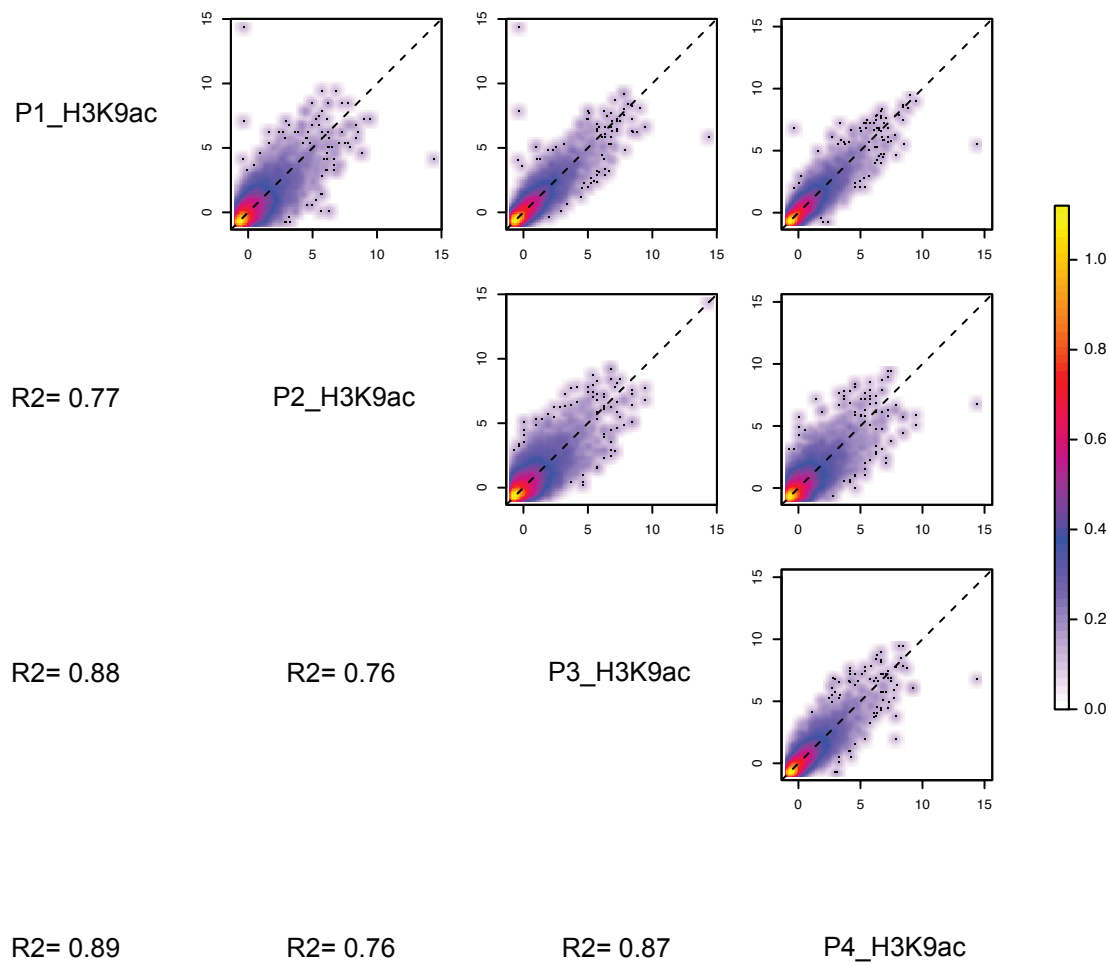

Supplement: Figure S2 — Correlation analysis for H3K27me3 and H3K9ac. A) H3K27me3 binding data from 4 donors was compared against each other with respect to binding to promoters. Quantile-normalized read counts per promoter interval (+/−1 kb around the transcriptional start sites) were plotted for all pair-wise combinations as smoothed scatterplots (color-coding of density is shown in color key). Furthermore Pearson’s R was calculated for each combination. B) Same as in A) for H3K9ac. (PDF) [file pone.0094164.s002.pdf]

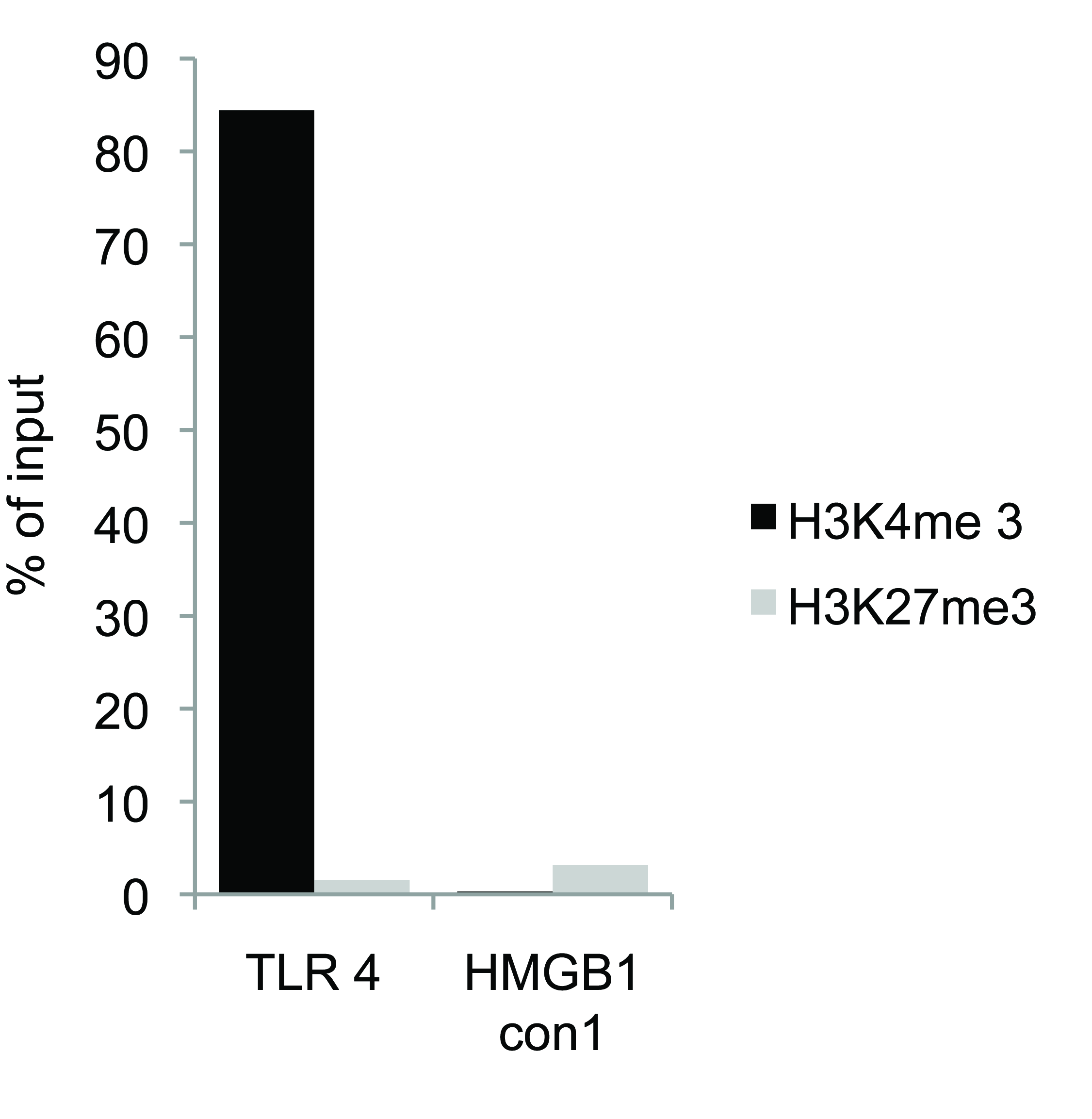

Supplement: Figure S3 — qPCR for validation of individual ChIP. Exemplary ChIP-qPCR for one of the analysed donors: analysis of H3K4me3 and H3K27me3 binding to the promoter of the TLR4 gene and a control region of the HMGB1 locus is shown. The TLR4 gene is active in CD14++ CD16- monocytes and accordingly we find strong association of H3K4me3 with the promoter. In contrast no H3K27me3 can be detected. As expected the control region in the HMGB1 locus (HMGB1 con1) is devoid of both histone modifications. (TIF) [file pone.0094164.s003.tif]
